# Full-visible-spectrum perovskite quantum dots by anion exchange resin assisted synthesis

**DOI:** 10.1515/nanoph-2021-0768

**Published:** 2022-02-18

**Authors:** Chenhui Wang, Junhu Cai, Yuanyuan Ye, Xinpei Hu, Lijuan Zhong, Hongxing Xie, Enguo Chen, Yun Ye, Sheng Xu, Jie Sun, Qun Yan, Tailiang Guo

**Affiliations:** National and Local United Engineering Laboratory of Flat Panel Display Technology, College of Physics and Information Engineering, Fuzhou University, Fuzhou 350108, P. R. China

**Keywords:** all-inorganic perovskite quantum dots, anion exchange resin, full visible spectrum, ion exchange, photoluminescent light-emitting diodes

## Abstract

Photoelectric properties of all-inorganic perovskite quantum dots (IPQDs) highly depend on their synthetic route. However, current synthetic processes of IPQDs are widely facing potential unsustainable issues of containing nonreusable and high-cost auxiliary materials. In this work, full-visible-spectrum IPQDs were successfully synthesized by an environmentally friendly ion-exchange approach with a renewable and low-cost anion exchange resin. Introducing anion exchange resin brings the improvement of both optical performance and surface morphology of the prepared IPQDs. The emission wavelength of IPQDs can be precisely controlled without changing their inherent crystal phase, and those IPQD’s single crystals with poor morphology and unstable structure are selectively removed. The photoluminescence quantum yield (PLQY) and the fluorescence lifetime of the three-primary-color IPQDs can be dramatically improved to 93.69, 89.99, and 65.03% and 71.3 ns, 22.2 ns, and 13.2 ns, respectively. Notably, the red-emitting PQDs at 622 nm exhibit a record high PLQY. By using the prepared IPQDs for photoluminescent color conversion, the three-primary-color light-emitting diodes (LEDs) provided high brightness and wide color gamut simultaneously. This study provides new ideas for the environmentally friendly and sustainable synthesis route of IPQDs, and it is expected to show great ambitions in the display field.

## 1 Introduction

Perovskite quantum dots (PQDs) is currently becoming one of the most promising photoelectric materials due to their advantages of high absorption capability, narrow full width at half maximum (FWHM), high photoluminescence quantum yield (PLQY), controllable composition, and size adjustable emission spectrum, etc. [1], [2], [3], [4], [5], [6], [7]. Their application has covered most of the photoelectric field, including light-emitting diodes (LEDs), solar cells, photodetectors, and laser, just to name a few [8], [9], [10], [11], [12], [13], [14], [15]. All-inorganic lead halide PQDs (CsPbX_3_, X = Cl, Br, I) not only have comparable excellent quality to traditional all-inorganic group II–VI and III–V QDs [16], but also have lower sensitivity to oxygen and water while compared with organic–inorganic hybrid PQDs [17], [18], [19]. The morphology and photoelectric properties of PQDs highly depend on their composition, and can be optimized by their halogen components [20, 21]. The halogens can be linked to the PQDs by different synthesis approaches, resulting in various performances. Therefore, exploring an appropriate synthetic route is of great significance in PQD studies [22], [23], [24], [25].

The introduction of ion exchange opens up a new route for the synthesis of full-visible-spectrum IPQDs and the adjustment of their photoelectric properties. So far, by using CsPbBr_3_ PQDs as a matrix, the adjustable spectral range is only limited from green to blue by cation-exchange reaction, and the spectral redshift is hard to achieve. In order to achieve full-visible-spectrum emission, the CsPbX_3_ PQDs are generally synthesized by anion-exchange reaction between single halide compound CsPbBr_3_ and magnesium methyl halide at 40 °C [26]. However, the phase separation after reaction causes poor stability, resulting in limitation of photoelectric properties in device applications [27]. Moreover, full-visible-spectrum PQDs can also be prepared by adjusting the ratio of different components of lead-halide compounds through hot-injection method or by using organic ligands containing different halogens through ligand exchange reaction [28, 29]. It is worth noting that there exist potential unsustainable issues with current synthetic routes of the full-visible-spectrum PQDs, because nonreusable and high-cost auxiliary materials are extensively used during their synthetic process. Therefore, an environmentally friendly synthetic route for full-visible-spectrum PQDs is urgently needed to meet the sustainable development in resources and environment.

In this study, an environmentally friendly, highly controllable, and low-cost synthetic route of full-visible-spectrum PQDs was proposed. The anion exchange resin was introduced into the synthesis of PQDs for the first time. High optical performance and good surface morphology are simultaneously achieved by this method, and nonluminescent impurities can be selectively filtered. The most important characteristic of this method is that the reaction rate accelerates with the increase of the concentration gradient. Concentration gradient refers to the difference in the concentration of halogen ions contained in different mediums [30]. In this experiment, the mediums are PQDs stock solution and ion exchange resin. A large number of halogen anions are contained in the high molecular framework of the anion exchange resin after simple treatment, which can ensure the efficient reaction [31]. The color of the solution can be directly observed during the reaction process, and the photoluminescence (PL) spectrum of the solution can also be measured anytime. The target emission wavelength is highly controllable and can be obtained as required. Moreover, the anion exchange resin is a kind of insoluble polymer material, neither soluble in water nor in organic solvents. This means that the ion exchange resin after reaction can be recycled and reused for the next-round synthesis of PQDs, so as to save resources and reduce costs.

The central wavelength of the PL spectrum of the PQDs synthesized by this method covers the whole visible spectrum (400–700 nm) with narrow FWHM (13–34 nm). The prepared PQDs show complete and orderly arrangement of crystal grains and exhibit high stability in the air. For device evaluation, the three-primary-color PQDs were separately mixed with polydimethylsiloxane (PDMS), and then packaged on ultraviolet (UV) light-emitting diode (LED) chips as the color conversion layer. These LEDs exhibiting good performance demonstrate the feasibility of this unique synthesis method and the potential application prospects in lighting or display field.

## 2 Results and discussion

### 2.1 CsPbBr_3_ PQDs based on anion exchange matrix

In this work, CsPbBr_3_ PQDs were synthesized using the most frequently reported hot-injection method [3, 5]. The temperature during thermal injection was strictly controlled at 180 °C ± 2 °C in order to obtain cubic perovskite crystals. This temperature does not exceed the boiling point of oleic acid (OA), which avoids the increase of defects and poor performance due to the reduction of ligands [32]. In addition to OA, oleylamine (OAm) was also used as the ligand on the PQDs’ surface to reduce the surface defect state. These organic ligands are more likely to fall off in a solvent environment with strong polarity, and causes agglomeration and quenching of PQDs [33]. Therefore, weak polar cyclohexane was used as the storage solvent in this experiment.

The cesium oleate precursor should be strictly isolated from air during preparation and storage, because cesium oleate is easily oxidized that would reduce the performance of the synthesized PQDs. The color of the solution can be intuitively observed to roughly determine whether the precursor is oxidized. As shown in Figure S1, the normally unoxidized precursor solution exhibits a white precipitate and colorless supernatant at room temperature, while the supernatant after oxidation becomes yellow-brown. The reaction time after the injection of the precursor solution should not be too long. Long reaction time would bring a larger particle size and more uneven distribution of the prepared PQDs, because a large number of crystal seeds will rapidly nucleate with a high reaction activity of precursor resulting in a smaller size [34]. In contrast, the final size of the particles will be larger with lower reaction activity of precursor.

### 2.2 Type conversion of anion exchange resin

**Figure 1:**
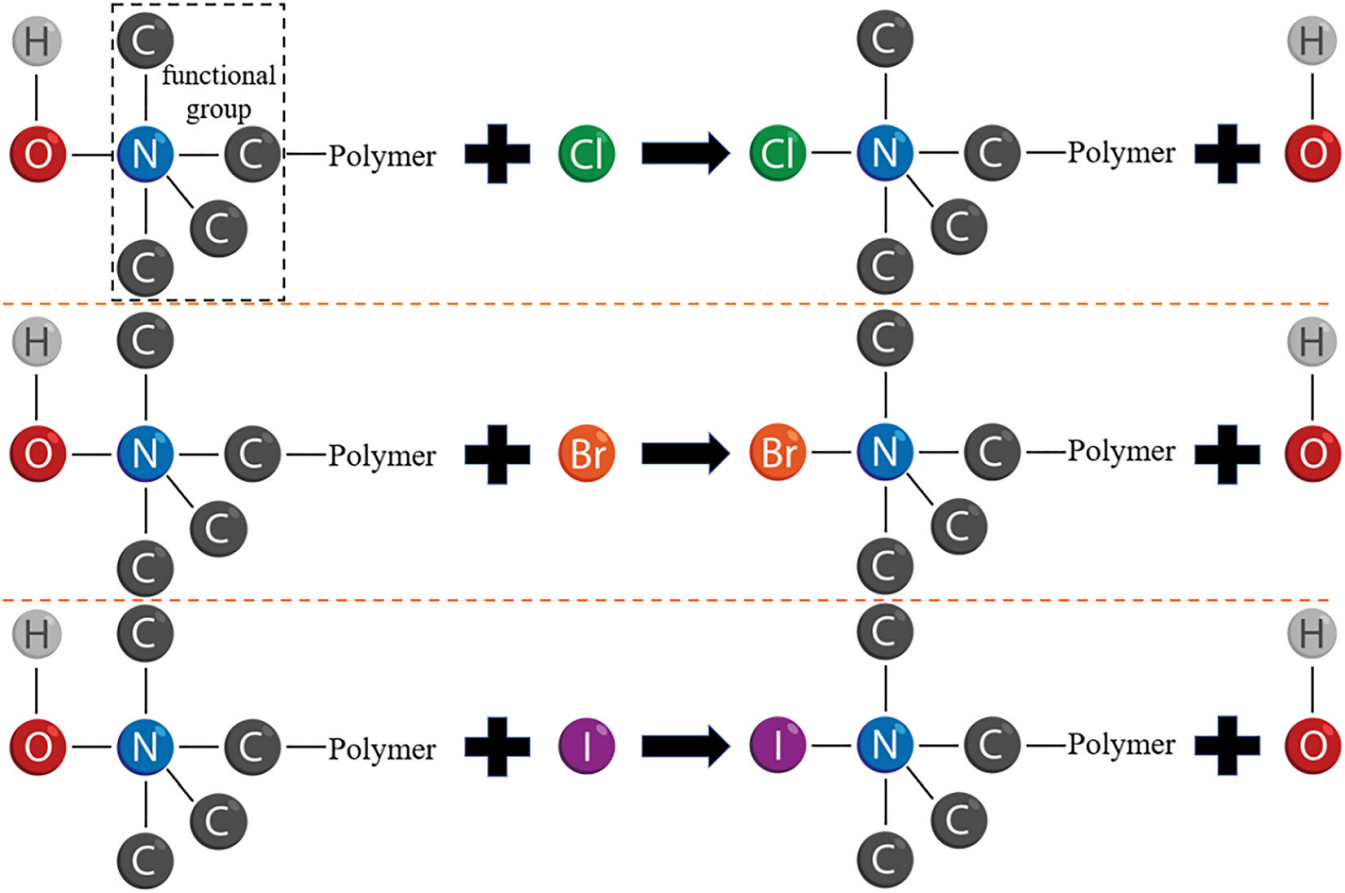
Schematic of type conversion reaction principle for anion exchange resin.

Type conversion of anion exchange resin is required before use to make the resin adsorb different halogen anions. This process only contains two simple steps. The anion exchange resin is firstly soaked in the saturated sodium salt aqueous solution containing the corresponding halogen, and then dried in oven. The type-Cl, type-Br, and type-I anion exchange resins obtained by type conversion and the corresponding images observed by scanning electron microscope (SEM) are shown in Figure S2. The chemical reaction principle of the type conversion is represented in Figure 1, where the polymer is formed by copolymerization of styrene and divinylbenzene. Moreover, the carbon atoms in the picture are all saturated, and the connected hydrogen atoms are not drawn in the picture.

This is a reversible chemical reaction between the ions in liquid and solid phases. Some ions in the liquid phase that are more preferred by the ion exchange resin will be adsorbed by the corresponding functional groups [35]. In order to maintain the electrical neutrality of the aqueous solution, the ion exchange resin has to release equivalent ions back into the solution [36, 37]. The saturated NaCl solution used in the experiment contains 61.4 mmol NaCl, the saturated NaBr solution contains 87.8 mmol NaBr, and the saturated NaI solution contains 122.6 mmol NaI. Halogen content is far greater than the total exchange capacity of 1 g anion exchange resin of 3.6 mmol. Therefore, the anion exchange resin after type conversion is considered without hydroxide ions. As shown in Figure S2, the appearance of type-Cl, type-Br, and type-I resins changes from light golden yellow to deep golden yellow.

### 2.3 Optical properties of PQDs by anion exchange resin

Type-Cl and type-I anion exchange resins were mixed with the CsPbBr_3_ PQDs solution in a fixed proportion, and the optical properties of the PQDs can be precisely controlled by reaction time. The exchange mechanism of ion exchange resin is similar to the exchange between lattice ions in crystals and electrolyte solution ions [38]. The ion exchange resin can be regarded as a kind of polyelectrolyte with large molecular weight. The ions combined with the functional groups in the ion exchange resin can be exchanged with certain ions in the electrolyte solution when in contact.

The ratio of resin to PQDs solution was fixed to 0.05 g/mL after several tentative experiments. In order to describe the ion-exchange reaction process more clearly, Figure S3 shows the single crystal structure of CsPbBr_3_ PQDs, where halogen atoms act as the skeleton of a regular octahedral structure. During the ion-exchange reaction process, the Cl^−^ and I^−^ adsorbed on the anion exchange resin were released in large quantities and exchanged with the Br^−^ existing in the original PQDs to convert CsPbBr_3_ into new compounds CsPbBr_*x*_I_3−*x*_ and CsPbBr_*x*_Cl_3−*x*_. The reaction conditions are so mild that it can proceed smoothly at room temperature. Since only the exchange between halogens occurs, nonluminescent impurities will not be introduced.

**Figure 2:**
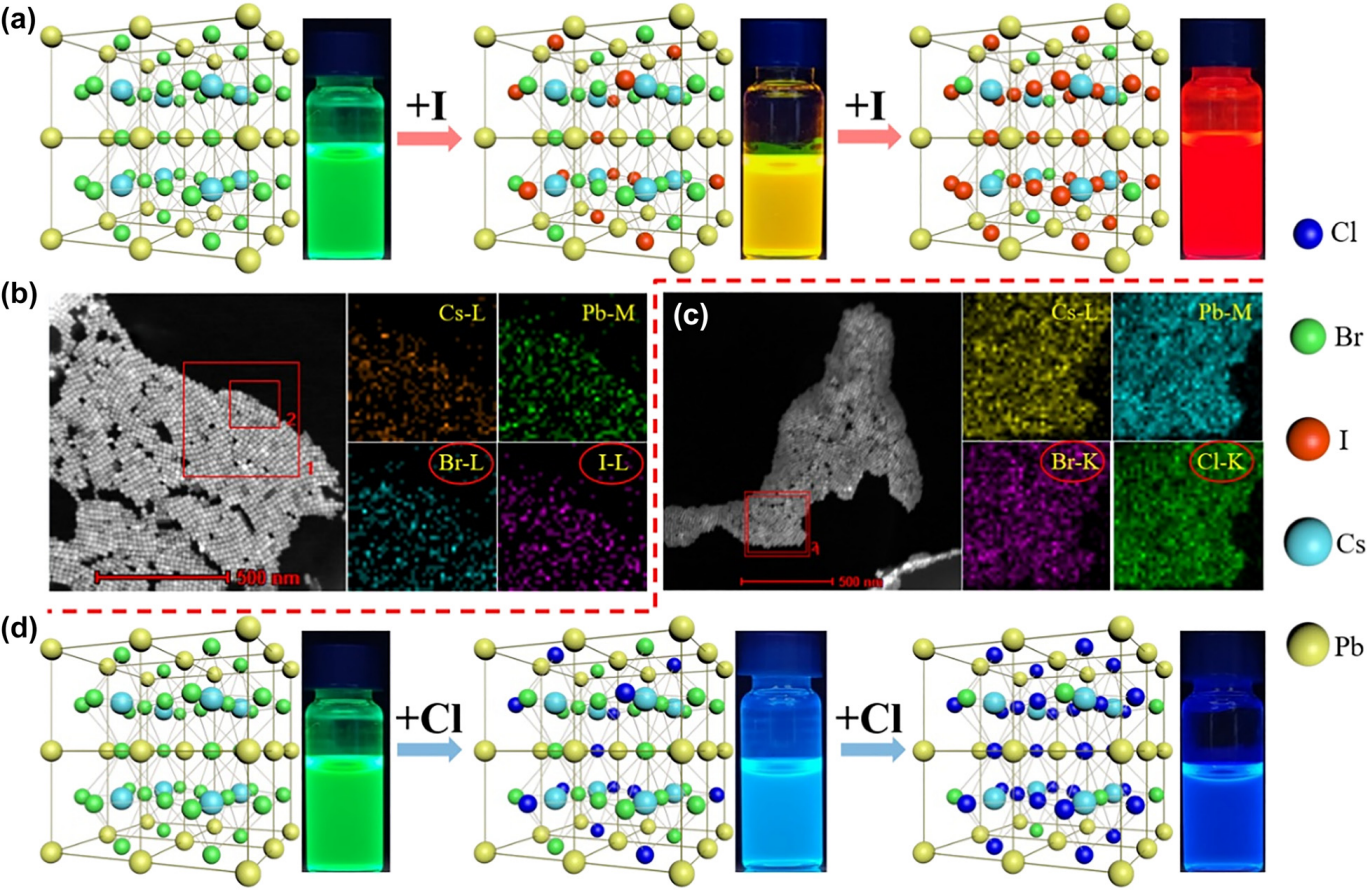
The crystal structures and composition changes of PQDs during the ion-exchange reaction. (a) Type-I anion exchange resin and (d) Type-Cl anion exchange resin participating in the reaction. Elemental mapping image of ion-exchange reaction products (b) CsPbBr_*x*_I_3−*x*_ and (c) CsPbBr_*x*_Cl_3−*x*_.

Figure 2a and d shows the crystal structures and compositional changes of PQDs during the ion-exchange reaction. The elemental mapping images of the CsPbBr_*x*_I_3−*x*_ and CsPbBr_*x*_Cl_3−*x*_ compounds produced by the reaction are shown in Figure 2b and c, which requires the help of the transmission electron microscope (TEM). The inner red box in Figure 2b and c is the observation area where the test is performed. It is worth mentioning that the energy of the electron beam needs to be carefully chosen for the elemental mapping test, because the resolution and the energy of the electron beam are positively correlated, and the halogen component contained in the PQDs will be quickly decomposed under the irradiation of high-energy electron beams so as to destroy its crystal structure. The results in Figure 2 show that this reaction does not change the original octahedral crystal structure of PQDs, but only provides the required replacement between halogens. The uniformity of the element distribution shows that Cs, Pb, Br, Cl, and I atoms were effectively and uniformly incorporated into the PQDs, proving the smooth process of the ion-exchange reaction. The corresponding results of EDX element analysis are shown in Figure S4. The weight percentage and atomic number percentage of the corresponding elements in the sample are summarized in Table S1. These results provide evidence for the uniformity of the element distribution.

A dynamic equilibrium eventually reaches due to the insolubility of the anion exchange resin and the reversible ion-exchange reaction. The ion-exchange reaction can be stopped anytime by separating the resin from the solution. That means the reaction time can be precisely controlled to obtain target emission wavelength of the synthesized PQDs. As reaction time increases, the PQDs solution will undergo a visible color change and exhibit bright fluorescence under the UV light excitation. Because of the difference in ion exchange ability between Cl^−^, I^−^ and Br^−^, the reaction between I^−^ and Br^−^ is more likely to occur than that between Cl^−^ and Br^−^, which brings the difference in reaction time [39].

**Figure 3:**
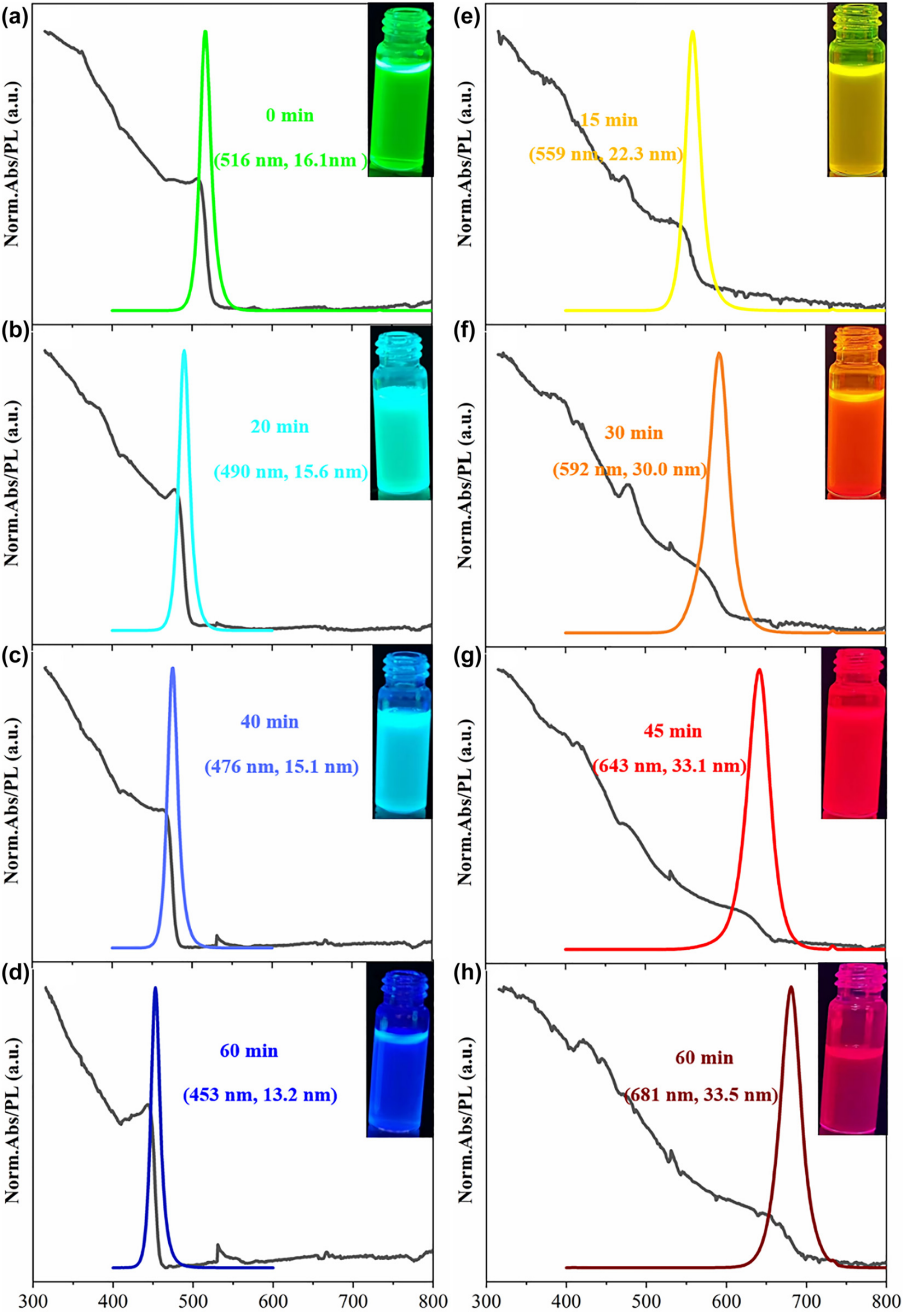
Absorption and PL spectra of the mixture of anion exchange resin and CsPbBr_3_ PQDs at different reaction times. (a)–(d) Type-Cl anion exchange resin participates in the reaction. (e)–(h) Type-I anion exchange resin participates in the reaction. Insets show the corresponding photographs.

It is found that the absorption and PL spectra of PQDs all change with the increase of reaction time. Figure 3a and e–h show the spectra during the reaction process of type-I anion exchange resin with CsPbBr_3_. The numbers in parentheses refer to the center wavelength and FWHM of the corresponding PL spectrum, respectively. The absorption spectra of PQDs broadened significantly with reaction time, which indicates the formation of CsPbBr_*x*_I_3−*x*_ compounds. At the same time, the corresponding PL spectrum shows a significant red shift. The center wavelength moved from 516 to 681 nm after reaction for 60 min, and meanwhile the FWHM widened from 16.1 to 33.5 nm. As shown in Figure 3a–d, the absorption spectra of PQDs gradually shrink with the reaction of type-Cl anion exchange resin with CsPbBr_3_. A narrowing of the absorption range occurs with the formation of CsPbBr_*x*_Cl_3−*x*_ compounds. In the meantime, the PL spectrum of PQDs has a significant blue shift. The center wavelength shifted from 516 to 453 nm after reaction for 60 min, and the FWHM shrunk from 16.1 to 13.2 nm.

There are two main factors affecting the PL center wavelength and FWHM of lead-halide PQDs. One is the particle size of the PQDs, and the other is the ratio of different halogen components in the compound [40, 41]. Generally, the emission wavelength of PQDs has a red shift with the increasing particle size. However, the impact of the particle size on the center wavelength is limited, and the overall variation range is within 40 nm. In Figure 3, it can be inferred that the large shift of the center wavelength is mainly caused by the change of the halogen composition. This also proves that I^−^ and Cl^−^ were successfully adsorbed on the anion exchange resin during the ion exchange process with Br^−^ in the CsPbBr_3_ PQDs.

It is significant to obtain narrow FWHM of PQDs during this reaction. The narrower the FWHM is, the higher uniformity the generated PQDs could provide. The FWHM is an important indicator for evaluating the monochromaticity of the material. Narrower FWHM means better monochromaticity of PQDs, and further determines the color characteristic in display applications.

Compared with other ion exchange methods to achieve full-visible-spectrum tunable PQDs, the proposed synthetic route based on anion exchange resin is environmentally friendly and more in line with the requirements of sustainable development in resources and environment. Theoretically, anion exchange resin can be reused almost indefinitely under mild conditions. The reusability of anion exchange resin was verified by experiment. Type-I anion exchange resin which has reacted with CsPbBr_3_ PQDs was taken out by filtration. As shown in Figure S5a, the surface of the reacted resin appears red due to the adhesion of CsPbBr_*x*_I_3−*x*_ PQDs. Then, the regenerated type-I anion exchange resin and CsPbBr_3_ PQDs were mixed again in the original ratio. The results in Figures S5 and S6 show that the ion-exchange reaction still has strong ion exchange capacity that can be reused for PQDs synthesis effectively.

### 2.4 Effect on surface morphology and fluorescence lifetime of PQDs

Apart from the adjustable PL emission, the ion-exchange reaction based on the anion exchange resin is also found to improve the surface morphology of PQDs to a certain extent. Oxidized precursor solution was used for preparing PQDs by the thermal injection method, and some CsPbBr_3_ PQDs with poor morphology were obtained for comparison, namely G-PQDs. By controlling the synthesis ratio of PbCl_2_, PbBr_2_, and PbI_2_, the CsPbBr_*x*_I_3−*x*_ and CsPbBr_*x*_Cl_3−*x*_ PQDs with poor morphology were synthesized, called R-PQDs and B-PQDs, respectively. Figure S7 shows the TEM images of R, G, and B-PQDs, respectively.

Subsequently, the previously prepared type-Cl, type-Br, and type-I anion exchange resins were added to the B-PQDs, G-PQDs, and R-PQDs solution in equal amounts respectively. The purification treatment was performed after predetermined reaction time. The treatment steps can be found in the experimental section. The red, green, and blue PQDs were finally obtained, called A-R-PQDs, A-G-PQDs, and A-B-PQDs, respectively. The letter “A” stands for anion exchange. The emission center wavelengths of them are 622, 512 and 457 nm, respectively.

**Figure 4:**
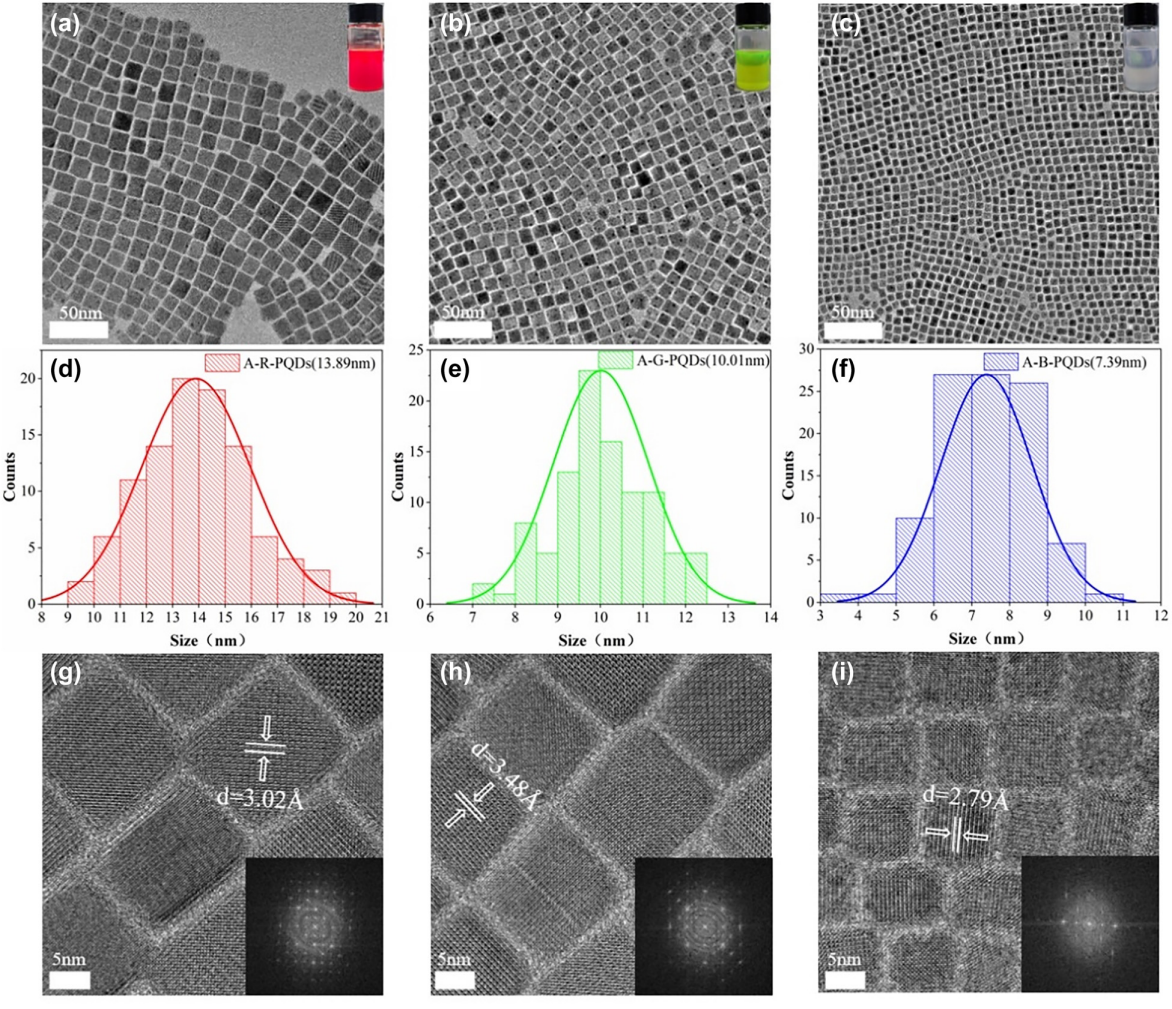
TEM images of (a) A-R-PQDs, (b) A-G-PQDs, and (c) A-B-PQDs; particle size distribution statistics of (d) A-R-PQDs, (e) A-G-PQDs, and (f) A-B-PQDs; high-resolution TEM images and crystal diffraction patterns of (g) A-R-PQDs, (h) A-G-PQDs, and (i) A-B-PQDs.

Figure 4a–c shows the TEM images of A-R-PQDs, A-G-PQDs, and A-B-PQDs, respectively. It can be seen intuitively that the PQDs after ion exchange show clear cubic phase, and the crystal grains are complete and neat. There is no surface damage or structural distortion, and lattice fringes can be observed clearly. Diffraction patterns can be observed in the high-resolution TEM images in Figure 4g–i, indicating that these PQDs have high crystallinity and crystal integrity [42], [43], [44]. Among them, A-G-PQDs are obtained by ion exchange between Br^−^ attached to the anion exchange resin and Br^−^ in the original G-PQDs. The final CsPbBr_3_ PQDs have no change in composition elements, proving that there are other reasons for the morphology improvement.

This phenomenon can mainly be attributed to the continuous release of excess halogen anions on the anion exchange resin. Selective removal of the PQDs with poor morphology can be achieved by introducing this anion exchange resin. The crystallinity of PQDs was enhanced by passivating surface defects. The excess ions and atoms on the surface of PQDs were dissolved and removed in a unique manner. The complete unit crystals were finally left in the solution. PQDs with poor morphology that has unstable crystal structure correspond to poor crystallinity, so that the ions and atoms are more easily adsorbed and removed by the functional groups on the ion exchange resin.

Figure 4d–f shows the particle size statistics of A-R-PQDs, A-G-PQDs, and A-B-PQDs through randomly selecting 100 crystal grains for size calculation. The average particle sizes are 13.89 nm, 10.01 nm, and 7.39 nm respectively, and the particle size distribution satisfies the normal distribution as expected. The X-ray diffraction (XRD) patterns corresponding to the TEM images also confirm the crystal characteristics of the PQDs. It can be seen from Figure S8 that all these PQDs have strong diffraction peaks near 15.1° and 30.5°. The peak position moves to a smaller angle with the red shift of the PL wavelength. These two diffraction peaks correspond to the (100) and (200) crystal planes of the cubic phase, respectively. Among them, the (200) crystal plane can also represent the second-order diffraction of the (100) crystal plane [45, 46]. All these results prove that the unique ion exchange method not only does not damage the morphology and performance of PQDs, but can also improve them to a certain extent. As another comparison, Figure S9 shows the TEM images of the PQDs prepared by unoxidized precursors and treated by anion exchange resin, which also well support the selective removal of the PQDs with poor morphology and unstable structure by the anion exchange resin.

In addition to the morphology improvement, the excess halogen ions during reaction can also help passivating surface defects on PQDs and reduce nonradiative recombination paths. This effect can be intuitively reflected by the change of fluorescence lifetime. The third-order decay exponential model was used to fit the fluorescence decay curve. Table S2 shows the fitting equations and the corresponding fitting parameters.

**Figure 5:**
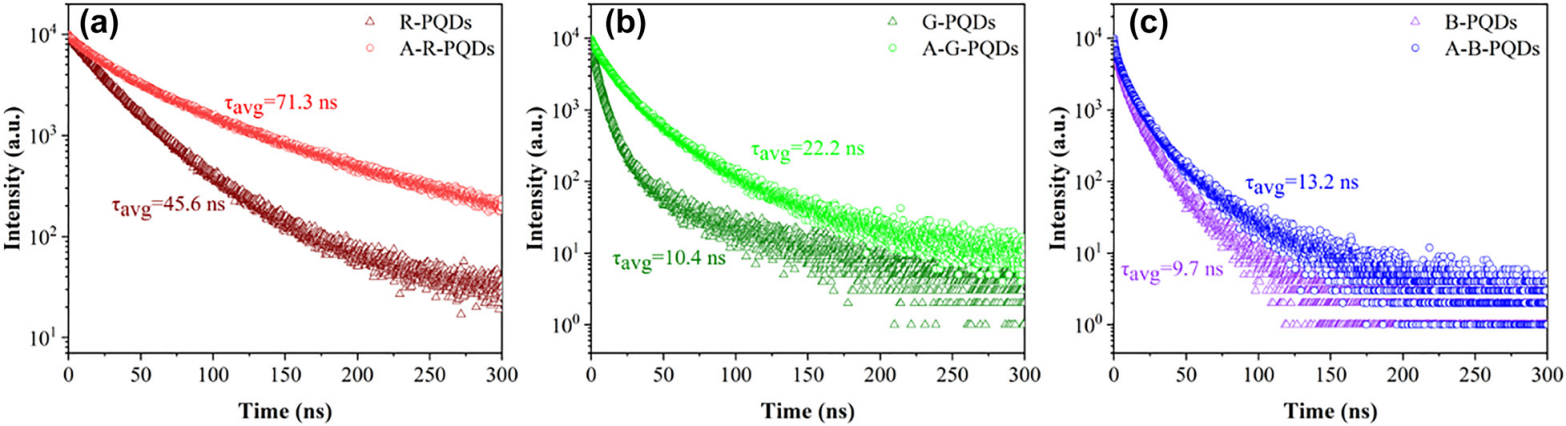
Comparisons of the TRPL between (a) R-PQDs and A-R-PQDs, (b) G-PQDs and A-G-PQDs, and (c) B-PQDs and A-B-PQDs.

Figure 5a–c compare the time-resolved photoluminescence (TRPL) spectra of the PQDs before and after reaction. It can be found that the average fluorescence lifetimes of PQDs increase from 45.6 to 71.3 ns, 10.4 nm to 22.2 ns, and 9.7–13.2 ns, respectively. In general, higher fluorescence lifetime means fewer surface defects [47]. Defects in the crystal can trap radiated photons and act as a center for nonradiative recombination to confine the stimulated emission process. This will lead to reduced emission and decreased average fluorescence lifetime of PQDs [48]. The fitting parameters *t*_1_ and *t*_2_ in Table S2 represent the corresponding lifetime for nonradiative process and radiative process, respectively. The increase in the average fluorescence lifetime indicates that the excess halogen anions released by the anion exchange resin play an important role in the passivation of the surface defect states of the PQDs, which not only enhances the stimulated radiation, but also weakens the unstimulated radiation. In other words, these excess halogen atoms are served as a self-passivation layer that effectively inhibits the capture of photo-induced carriers by surface defects. This is therefore beneficial to improve the stability and fluorescence efficiency of PQDs [49].

Similar to fluorescence lifetime, the PLQY is also significantly improved. Figure S10 shows the original spectral data of the measured PLQY. Equation (1) is the calculation method, where $I_{\text{em}}^{\text{PQDs}}\left(\lambda \right)$ and $I_{\text{em}}^{\text{ref}}\left(\lambda \right)$ represent the emission intensity of the samples with and without PQDs, and $I_{\text{ex}}^{\text{PQDs}}\left(\lambda \right)$ and $I_{\text{ex}}^{\text{ref}}\left(\lambda \right)$ are the integrated excitation intensity of the samples with and without PQDs, respectively. Here, the test sample is diluted PQD solution, and a pure cyclohexane solution is used as a reference equaling in volume to the test sample.

(1) $$\text{PLQY}=\frac{\text{photons emitted}}{\text{photons absorbed}}=\frac{\int \left(\frac{\lambda }{hc}\right)\times \left[I_{\text{em}}^{\text{PQDs}}\left(\lambda \right)-I_{\text{em}}^{\text{ref}}\left(\lambda \right)\right]\text{d}\lambda }{\int \left(\frac{\lambda }{hc}\right)\times \left[I_{\text{ex}}^{\text{ref}}\left(\lambda \right)-I_{\text{ex}}^{\text{PQDs}}\left(\lambda \right)\right]\text{d}\lambda }$$

Compared with R, G, B-PQDs, the PLQY of A-R, A-G, and A-B-PQDs increased from 68.82–93.69%, 53.23–89.99%, and 54.23–65.03%, respectively. This significant improvement in PLQY can be attributed to two main reasons. One is that the direct band gap characteristics of the material itself can increase the light absorption coefficient and accelerate the rate of radiation recombination [50]. The other important reason is the passivation effect of excessive halogen anions on the surface defects of the PQDs. In addition, the effect of solvent polarity on the ion exchange reaction rate was thoroughly explored in this experiment, which can be found in Figure S11 and the corresponding discussion.

A control group prepared by the common anion exchange process was set up to further demonstrate the superiority of anion-exchange resin-assisted synthesis of PQDs. Figure S12 shows the ion exchange reaction process, the TEM images and TRPL spectra of the resulting PQDs. These results well support that the anion exchange resin assisted synthesis is better on improving the morphology and quality of PQDs.

### 2.5 Application in color converted LEDs

Ion-exchange reaction based on anion exchange resin brings excellent optical properties of the PQDs. These PQDs are competitive alternative for color conversion in direct-view LED displays [51]. In order to achieve high-color-purity LED displays, the three-primary-color PQDs with emission wavelengths of 648 nm, 523 nm, and 441 nm were prepared. Although the central wavelength largely determines the color gamut of the display devices, it may bring potential ocular health issues, especially when the central wavelength falls into the blue hazardous region of the human eye. Moving the central wavelength of the color conversion material towards 465 nm is one of the common approaches in current display industry to minimize the blue light hazards [52]. This can also be realized by our synthesized PQDs due to their excellent spectral tunability. In this experiment, the three-primary-color PQDs were mixed with polydimethylsiloxane to prepare the color conversion layers for bare UV LEDs. The detailed preparation process has been listed in the experimental section. The absorption coefficients of the color conversion layers are 0.46 cm^−1^ for blue, 0.78 cm^−1^ for green, and 0.65 cm^−1^ for red under 365 nm UV light excitation, respectively. The PLQY of these layers fluctuates between 40 and 60% with the same thickness of 0.95 mm.

**Figure 6:**
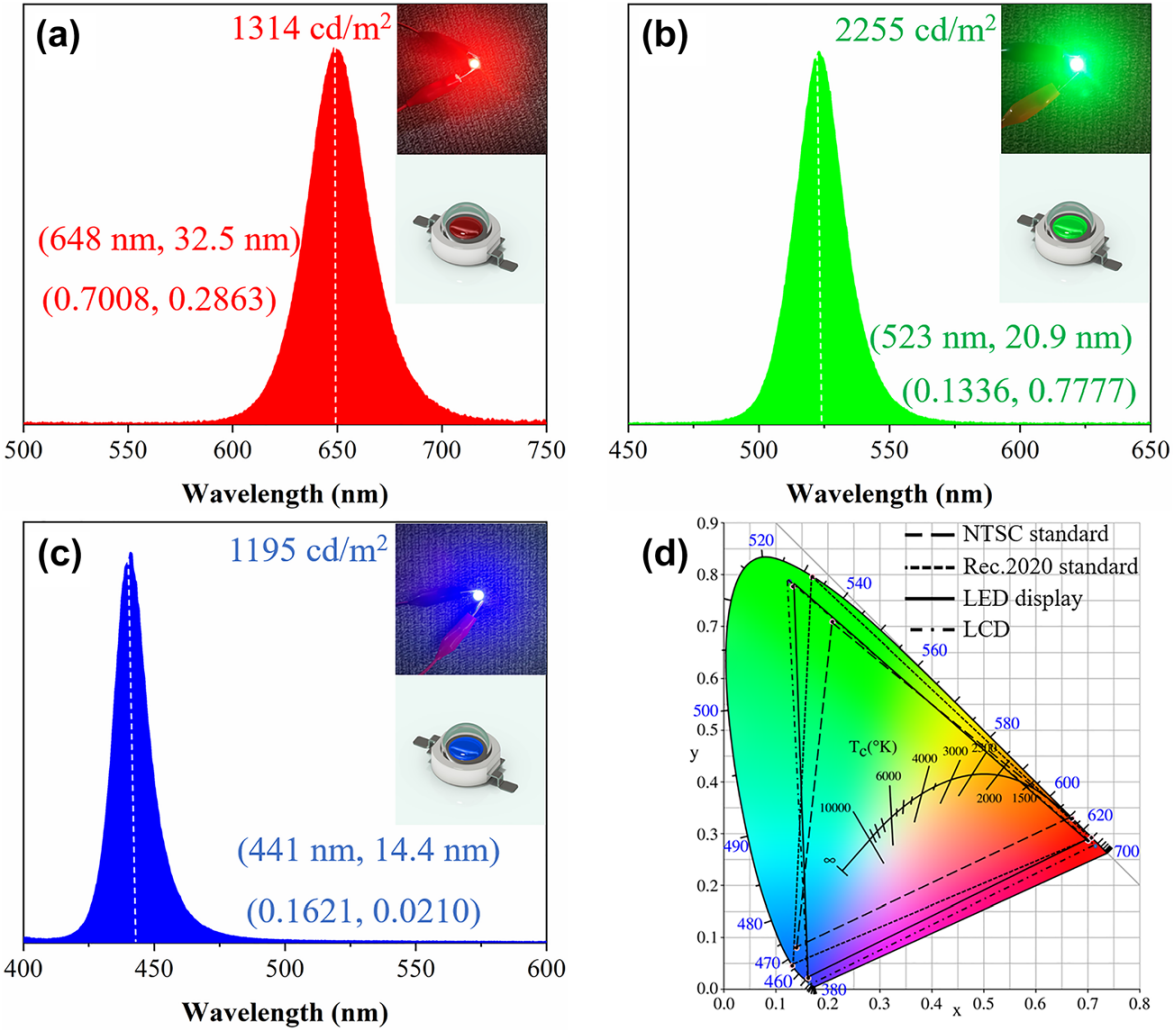
(a)–(c) The spectra of the three-primary-color LEDs and their working photographs; (d) the CIE chromaticity diagram of the direct-view LED display and the LCD compared with the NTSC and Rec. 2020 standard.

Figure 6a–c shows the spectra of the three-primary-color LEDs and the actual pictures when lighting up, respectively. The voltage was set to 3.4 V and the current was 0.2 A. The relative position of the LED and the luminance meter was fixed with the field of view of 0.2°. The brightness of the green LED reaches 2255 cd/m^2^, and the brightness of the red and blue LEDs is 1314 cd/m^2^ and 1195 cd/m^2^, respectively. The luminous efficacy of red, green, and blue LEDs is measured to be 59.3 lm/W, 78.5 lm/W, and 48.8 lm/W, respectively. Here, the luminous efficacy is defined as the ratio between the luminous flux (in lumens) and the input electrical power used to produce that luminous flux (in watts, W). Although the luminous efficacy reaches the average level of LEDs based on PQD materials, it still has much room to improve compared with cadmium QD-based LEDs [53], [54], [55]. The color coordinates and other parameters are listed in Table S3. Figure 6d shows the chromaticity coordinates of the prepared LEDs under Commission Internationale del’Eclairage (CIE) 1931 chromaticity diagram. For a more comprehensive comparison, both the National Television Standards Committee (NTSC) standard and ITU-R Recommendation BT. 2020 (Rec. 2020) RGB primaries were used here to discuss the color gamut of the three-primary-color LED display. The triangles composed of solid line, long dash line, and short dash line represent the color gamut range of the LED display, 100% NTSC standard, and 100% Rec. 2020 standard, respectively. The corresponding color coordinate parameters are listed in Table S4. The color gamut reaches 131.22% under NTSC standard and 98.06% under Rec. 2020 standard. It demonstrates the potential application prospects in the display field in the future [56, 57].

The above results have discussed the application scenario of direct-view LED display based on the prepared color converted LEDs. While these LEDs are used as the backlit sources of liquid crystal display (LCD), the color gamut will strongly depend on the color filter quality of the LCD. That is to say, the transmittance of the color filters has to be considered. Figure S13a shows the transmittance curves of currently commercialized color filters for three primary colors, and Figure S13b–d show the three-primary-color spectra after passing through the color filters for the LCD. Due to the light absorption and filtering effect, the FWHM of blue and green spectra is slightly shrunk, while the FWHM of the red spectrum is slightly broadened. The central wavelength of the three spectra remains unchanged. Overall, the monochromatic coordinates and color gamut of the LCD are affected by both the spectra of the LED backlight and the transmittance of the color filters. The resulting color triangle is represented by a dot dash line in Figure 6d. After calculation, the color gamut of the LCD reaches 138.52% under NTSC standard and 103.51% under Rec. 2020 standard. It means that the color gamut of the LCD can be slightly improved because of the spectral shaping effect by the color filters.

The changes in the PL spectrum and light intensity of the color-converted LEDs are recorded when the operating current gradually increases and the voltage is kept unchanged. As shown in Figure S14a–c, the bathochromic shift and FWHM broadening of the PL spectrum are observed with the operating current increase from 200 to 350 mA for three color converted LEDs. The reason is that the LED temperature gradually increases with the operating current, and high working temperature will lead to the crystal phase separation and grain agglomeration of the PQDs. In Figure S14d, it is observed that the light intensity first increases and then decreases with the increase of the operating current. The increase of the operating current can enhance the excitation of PQDs and thus increase the light intensity. However, too high temperature instead causes the decomposition of PQDs and further weaken the LED’s light intensity.

The operational lifetime of the fabricated LEDs is evaluated. The UV LED itself can provide the operational lifetime higher than 30,000 h, so that the effective operational lifetime of the color converted LEDs is dependent on the PQD layer. In this experiment, the operating voltage and current of the LEDs were set to 3.4 V and 200 mA, respectively. The light intensity change of the fabricated LEDs was monitored continuously to obtain their operational lifetime. The measured results are shown in Figure S15, where the dotted line represents the effective operational time when the light intensity drops to half of the initial value, namely T_50_. The measured T_50_ of red, green, and blue LEDs is 7.7 min, 15.1 min, and 4.9 min, respectively. Due to the instability, PQDs are prone to phase separation under high temperature and long-term UV light excitation, leading to fluorescence quenching and light intensity decrease. The final effective operational lifetime of the red, green, and blue LEDs is 14.2 min, 22.1 min, and 10.0 min, respectively. At this point, the light intensity coming from the color conversion of PQDs drops to 0.

## 3 Conclusions

In summary, full-visible-spectrum tunable PQDs using CsPbBr_3_ as the matrix were successfully prepared through ion-exchange reaction with anion exchange resin. The unique characteristic of this synthetic route is environmentally friendly, highly controllable, and low cost, which matches the requirement of social sustainable development in resources and environment. The emission wavelength can be determined by the reaction rate that can be finely controlled by the amount of resin, reaction time, and solvent polarity. It is also discovered that the excess halogen anions released by the anion exchange resin can improve the morphology of PQDs and passivate the surface defects that brings an increase in the average fluorescence lifetime and PLQY. Compared with traditional hot-injection method, the fluorescence lifetime of the three-primary-color PQDs after ion exchange reaction increased to 71.3 ns, 22.2 ns, and 13.2 ns, respectively. Meanwhile, the PLQY reached 93.69, 89.99, and 65.03%, respectively. Notably, the red-emitting PQDs at 622 nm exhibit a record high PLQY. The superiority of this method to the common anion exchange method has also been demonstrated. The prepared three-primary-color PQDs were encapsulated with PDMS for color converted LEDs. The maximum monochromatic brightness can reach higher than 4000 cd/m^2^, and the corresponding luminous efficacy is higher than 78 lm/W. Wide color gamut reaching 131.22% NTSC (98.06% Rec. 2020) are achieved for the application scenario of direct-view LED display. While used as LCD backlight sources, the color gamut can further be expanded to 138.52% NTSC (103.51% Rec. 2020). It is expected that this approach will open up a new sustainable route for the synthesis of nanomaterials in the near future.

## Supplementary Material

Supplementary Material
