# Supplementary material for: Full-visible-spectrum perovskite quantum dots by anion exchange resin assisted synthesis
Source: Nanophotonics. 2022 Feb 18;11(7):1355–66. doi: 10.1515/nanoph-2021-0768 (PMC11501922; doi:10.1515/nanoph-2021-0768)
Supplement: Supplementary file 1 — Supplementary Material [file j_nanoph-2021-0768_suppl.docx]

Supplementary Material

**Full-visible-spectrum perovskite quantum dots by anion exchange resin assisted synthesis**

Chenhui Wang^†^, Junhu Cai^†^, Yuanyuan Ye, Xinpei Hu, Lijuan Zhong, Hongxing Xie, Enguo Chen^*^, Yun Ye, Sheng Xu, Jie Sun, Qun Yan, and Tailiang Guo

^†^These authors contributed equally to this work.

*Email: [ceg@fzu.edu.cn](mailto:ceg@fzu.edu.cn)


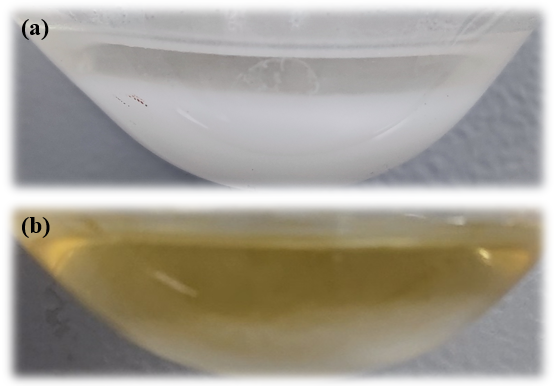


**Figure S1:** Cesium oleate precursor solution under (a) unoxidized state and (b) oxidized state.

It is clearly observed from Figure S1 that the cesium oleate precursor solution becomes light yellow after being oxidized. The reason is the oleic acid contained in the composition of this solution.


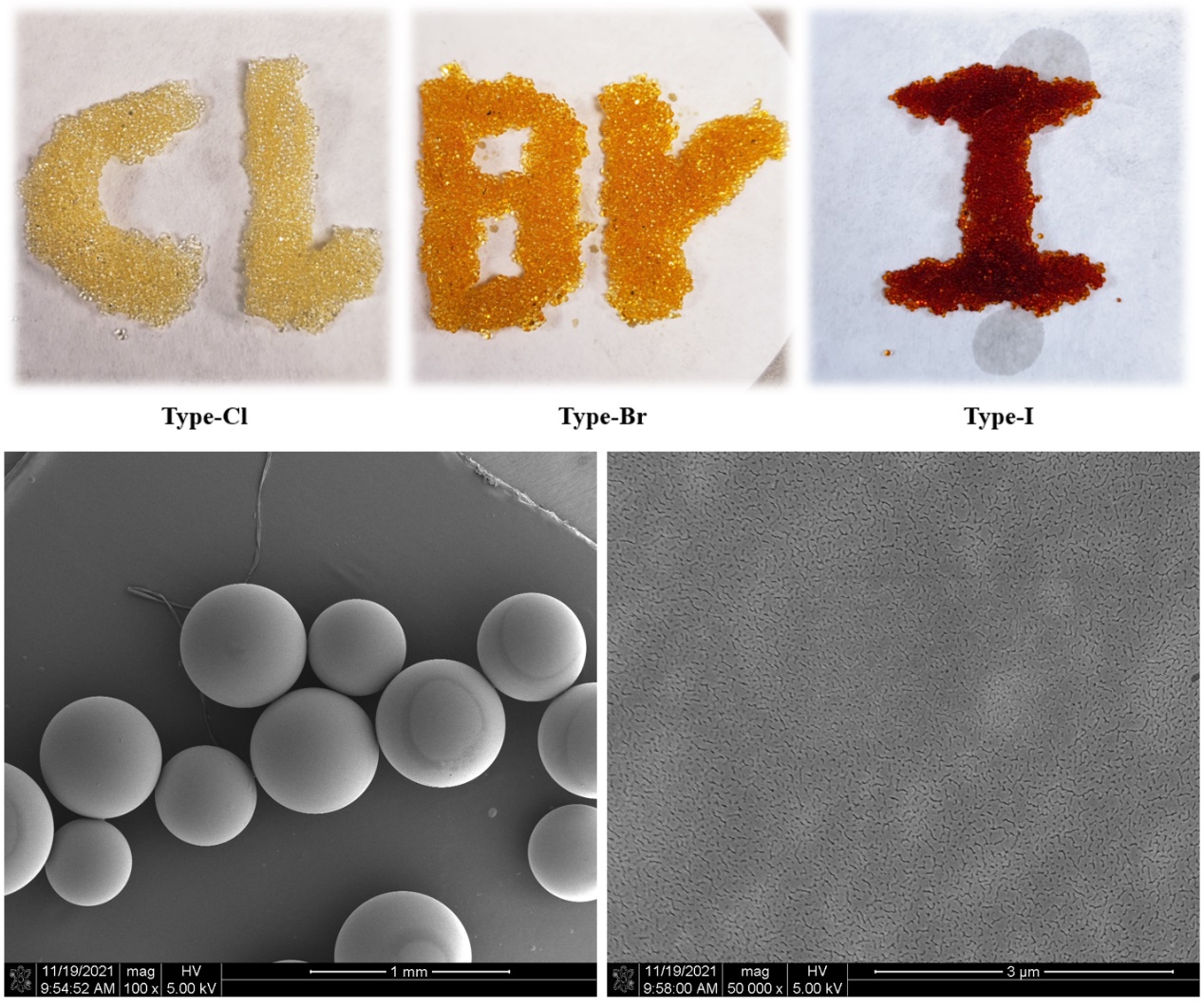


**Figure S2:** Different types of anion exchange resins after type conversion and the corresponding SEM images.

In Figure S2, the resin used in the experiment is macroporous strong basic anion exchange resin. The inert polymer network skeleton of this resin is formed by copolymerization of styrene and divinylbenzene. A large number of spongy micropores on the surface and inside provide good contact conditions for the ion exchange reaction. The functional group is quaternary ammonium group (-N(CH_3_)_3_). The appearance is light yellow to golden yellow spherical particles. The factory type is hydrogen-oxygen type. The total exchange capacity is about 3.6 mmol/g. The particle size ranges from 0.3 mm to 1.5 mm, and the pH value ranges from 0 to 14 can be used normally.


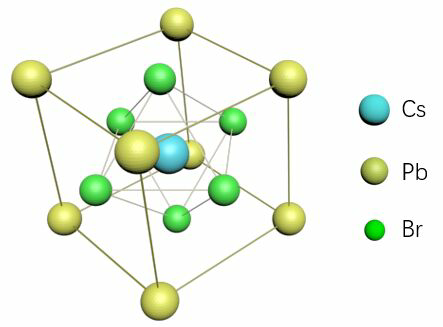


**Figure S3:** The octahedral structure of a single crystal of CsPbBr_3_ PQDs.


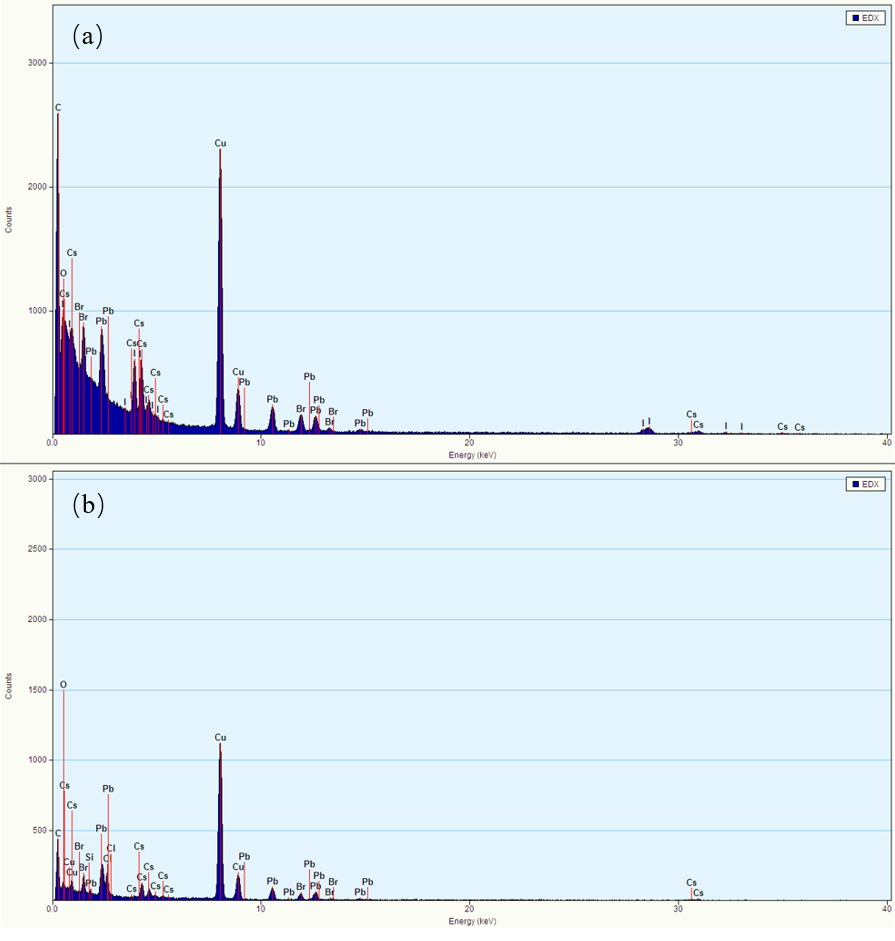


**Figure S4:** Elemental analysis by EDX on (a) CsPbBr_x_I_3-x_ and (b) CsPbBr_x_Cl_3-x_.


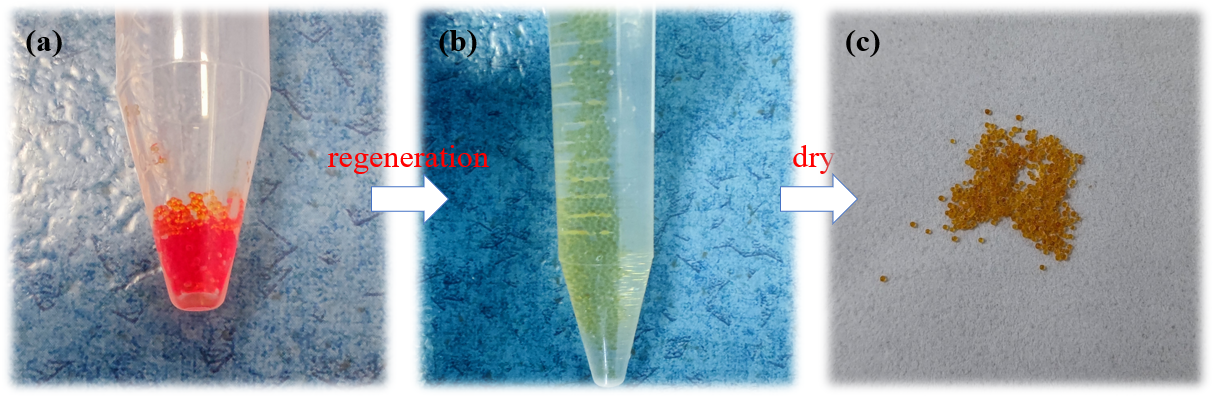


**Figure S5:** Regeneration of Type-I anion exchange resin. (a) Type-I anion exchange resin after reaction. (b) Mixture of anion exchange resin and NaI solution. (c) Type-I anion exchange resin after regeneration.


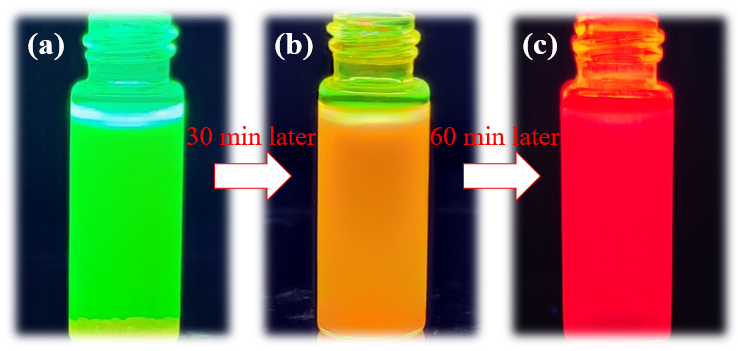


**Figure S6:** Reusability verification of the regenerated anion exchange resin. (a) Mixture of CsPbBr_3_ PQDs and regenerated Type-I ion exchange resin. (b) Reaction product after 30 min. (c) Reaction product after 60 min.


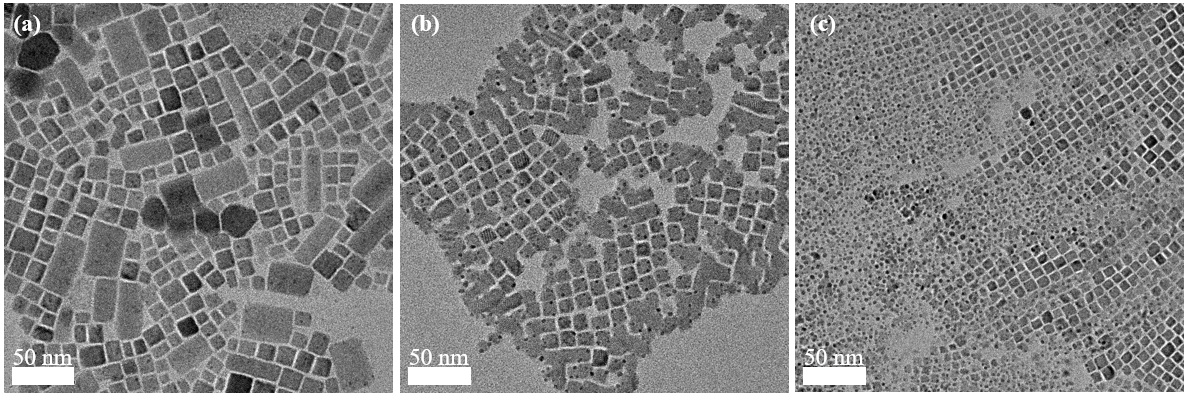


**Figure S7:** TEM images of (a) R-PQDs, (b) G-PQDs, and (c) B-PQDs.

As shown in Figure S7, there are many indications of the poor surface topography. It can be manifested by the huge difference in particle size. The grains at the edge are cohesive and the inner grains are arranged loosely and irregularly. Some of the grains are not even crystallized into a cubic phase.





**Figure S8:** XRD patterns of A-R-PQDs, A-G-PQDs, and A-B-PQDs.


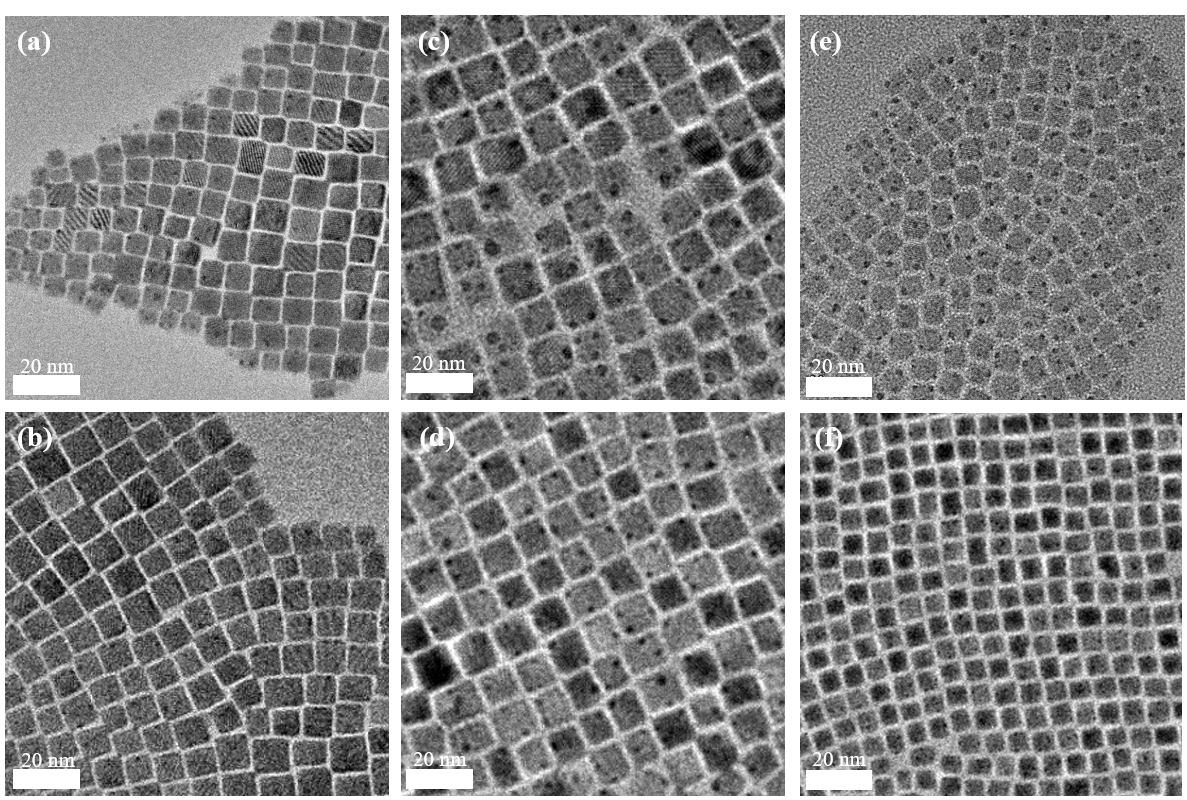


**Figure S9:** TEM images of (a) r-PQDs and (b) A-r-PQDs, (c) g-PQDs and (d) A-g-PQDs, (e) b-PQDs and (f) A-b-PQDs.

In this experiment, oxidized precursor solution was replaced by unoxidized one for preparing PQDs by the thermal injection method. All experimental procedures were kept the same. The synthesized three compounds were named as r-PQDs, g-PQDs, and b-PQDs, respectively. And then, they were treated with different types of resins, and the resulting products were named as A-r-PQDs, A-g-PQDs, and A-b-PQDs, respectively.

Figure S9 shows the TEM images of these compounds. Compared with the PQDs prepared by oxidized precursors and shown in Figure S7, the r-, g-, and b-PQDs in Figure S9 already show significantly better surface morphologies with no obvious structural distortion. However, there still exists uneven particle size distribution and irregular grain arrangement. After being treated with anion exchange resin, the surface morphologies were further improved with uniform particle size and regular grain arrangement. Moreover, the black spots generated by electron beam bombardment seem to be reduced, indicating that the crystallites remaining in the solution have a more stable crystal structure. These phenomena prove the selective removal of the PQDs with poor morphology and unstable structure by the anion exchange resin.


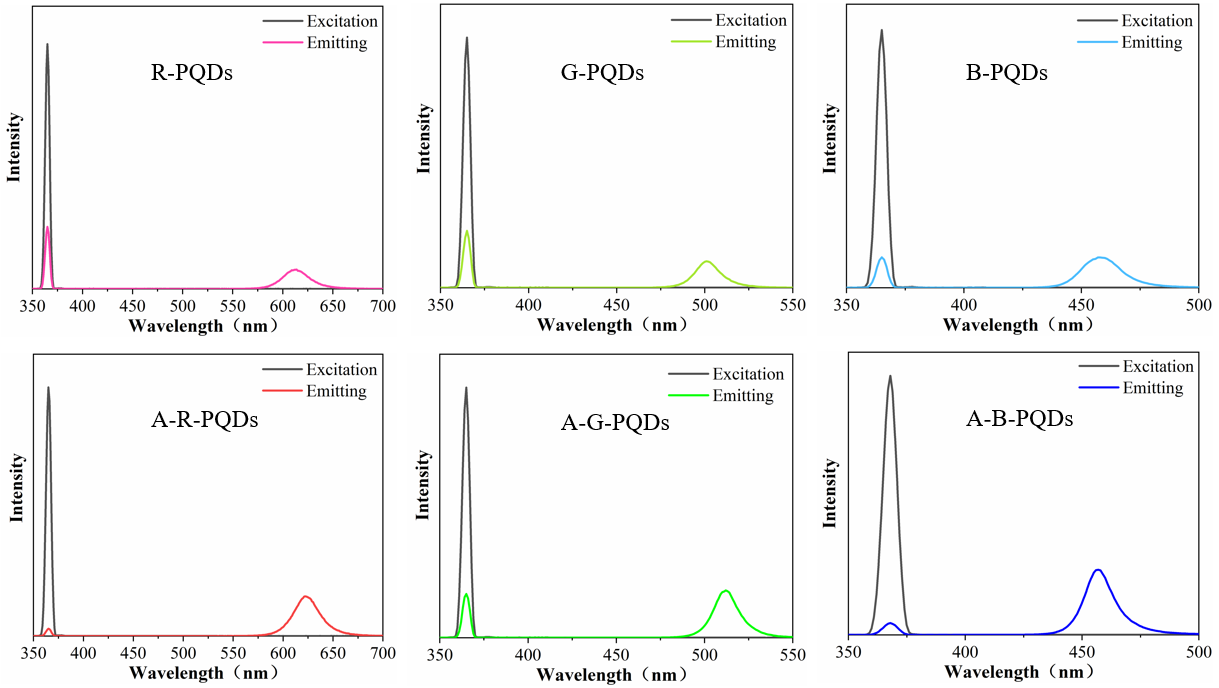


**Figure S10:** Original spectra of the measured PLQY.


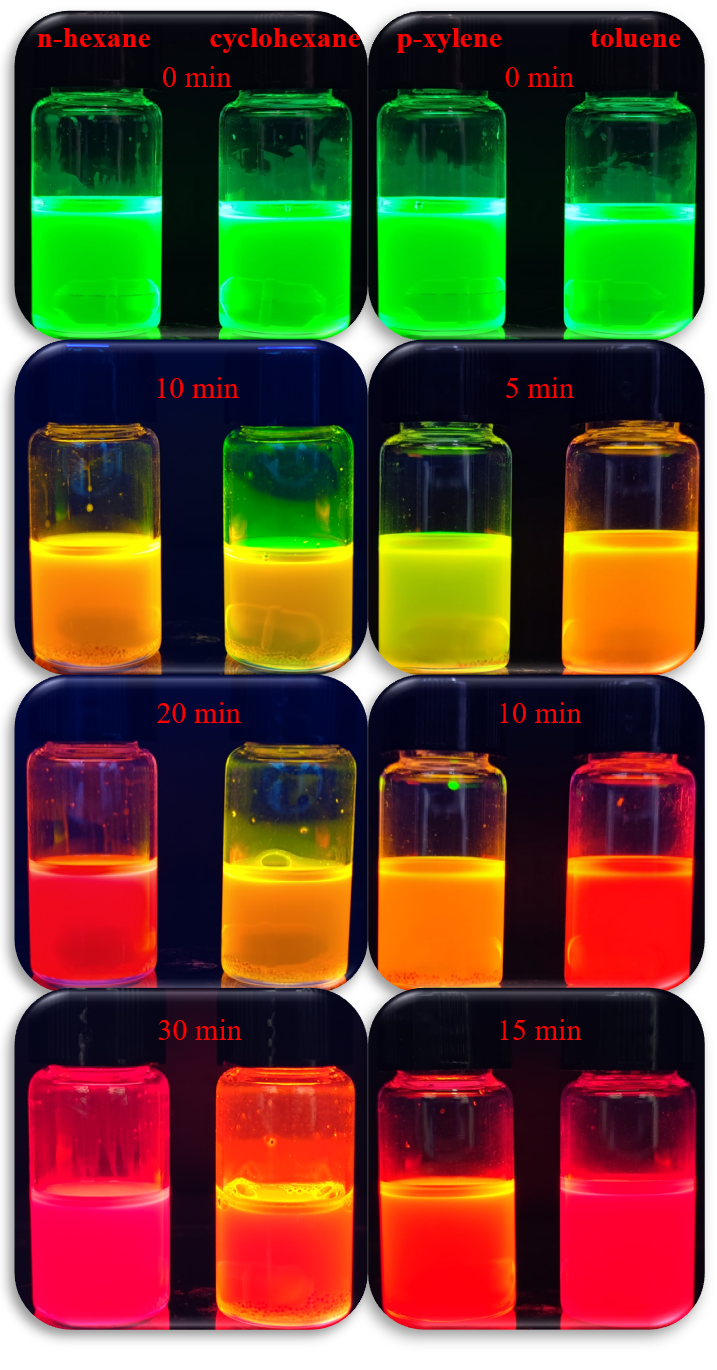


**Figure S11:** Comparisons of the ion exchange reaction results among the mixture of Type-I anion exchange resin and PQDs solutions with different solvents.

It is found in this experiment shown in Figure S11 that the ion-exchange reaction rate based on anion exchange resin can be greatly affected by the polarity of the dispersion solvent of PQDs. The larger the polarity of the solvent is, the faster the ion exchange rate is. The non-coincidence of the positive and negative charge centers in the molecule results in the uneven and asymmetrical charge distribution from the perspective of the entire molecule. Such molecules are called polar molecules. We chose toluene, p-xylene, n-hexane, and cyclohexane (the polarity intensity decreases sequentially) as the dispersing solvents of CsPbBr_3_ PQDs respectively, and used Type-I anion exchange resin and PQDs for ion exchange for verification.

The ratio of the resin to the PQDs solution was fixed at 0.05 g/mL, and the reaction was accelerated by magnetic stirring. Figure S10 shows the comparisons of the four solvents under different reaction times under the excitation of the 365 nm UV lamp. Obviously, under the same reaction time, the rate of ion-exchange reaction of PQDs dissolved in toluene and n-hexane is faster than that in p-xylene and cyclohexane, respectively. And overall, benzene reacts faster than hexane. The reason is that PQDs is a kind of ionic compound, and Br^-^ and I^-^ in the form of ions are affected by the dipole moment. Therefore, the polarity of the solvent becomes the driving force of the ion-exchange reaction.

Based on this, we only need to choose a solvent with a larger polarity to accelerate the ion-exchange reaction. However, from the perspective of long-term storage of PQDs, a larger solvent polarity will become an obstacle. In a solvent with larger polarity, the ligands on the surface of the PQDs are more likely to fall off, leading to agglomeration and fluorescence quenching of the PQDs. And due to the larger radius of I^-^, the weak bond strength between Pb and I will reduce the decomposition energy of PQDs [S1], which will make the CsPbBr_x_I_3-x_ compound easier to decompose in a strong polar solvent.


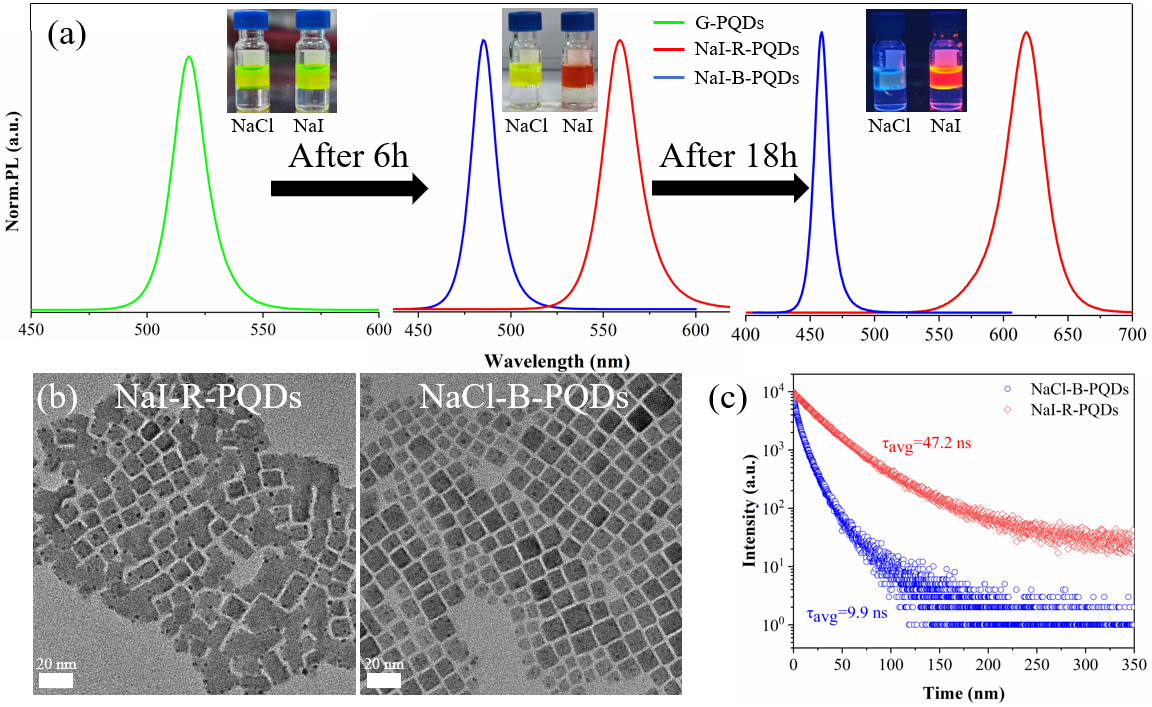


**Figure S12:** (a) Common anion exchange process, (b) TEM images of NaI-R-PQDs and NaCl-B-PQDs, (c) TRPL spectra of NaI-R-PQDs and NaCl-B-PQDs.

It is very essential to add control samples prepared by the common anion exchange process. The experimental results can well support that the anion exchange resin assisted synthesis is better on improving the morphology and quality of PQDs.

Figure S12a shows the the ion exchange reaction process by mixing the G-PQDs with saturated NaI and NaCl solutions, and then keeping for stratification. It can be observed that the ion exchange reaction would proceed spontaneously at the stratified interface. The resulting products were named as NaI-R-PQDs and NaCl-B-PQDs, respectively. Since the ion-exchange reaction occurs only at the layered interface, the diffusion rate of the product into the solution was quite slow, as well as the overall reaction rate. As shown in Figure S12a, the central wavelength moves from 518 nm to 485 nm and 560 nm after 6 h, and finally reaches 454 nm and 618 nm after 18 h.

Figure S12b shows the TEM images of NaI-R-PQDs and NaCl-B-PQDs. The cohesion of the outer grains and the loose arrangement of the inner grains are clearly observed, which is similar to the TEM image of G-PQDs shown in Figure S7b. The TRPL spectra of NaI-R-PQDs and NaCl-B-PQDs are drawn in Figure S12c. The third-order exponential model was also used here for fitting and calculation. The calculation results show that the average fluorescence lifetimes are 47.2 ns and 9.9 ns for NaI-R-PQDs and NaCl-B-PQDs, respectively. Compared with the results of R-PQDs and B-PQDs shown in Figure 5a and 5c, there is only minor improvement of 1.6 ns and 0.2 ns for NaI-R-PQDs and NaCl-B-PQDs, respectively. It indicates that the common anion exchange process cannot effectively passivate the surface defects of PQDs and improve the fluorescence lifetime.

In summary, the common ion-exchange reaction at the layered interface can introduce different halogens into PQDs for the purpose of spectral shift, but the reaction process is slow and not easy to achieve precise wavelength control. Moreover, the surface defects of PQDs cannot be passivated by excess halogen at the interface, and the grains with unstable structure and poor morphology cannot be selectively removed by the common anion exchange process. As a control experiment, the above results well support that the anion exchange resin assisted synthesis is better on improving the morphology and quality of PQDs.


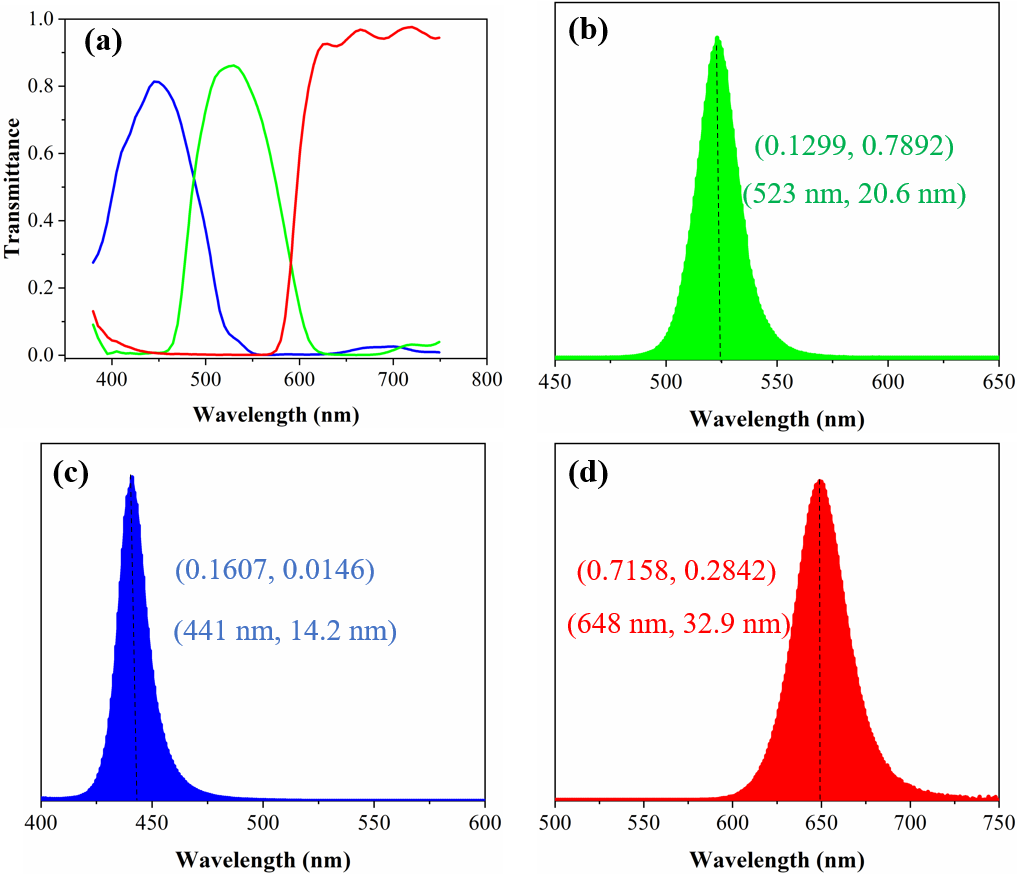


**Figure S13:** (a) Transmittance of commercialized color filters for three primary colors, (b)-(d) three-primary-color spectra passing through the color filter of LCD.


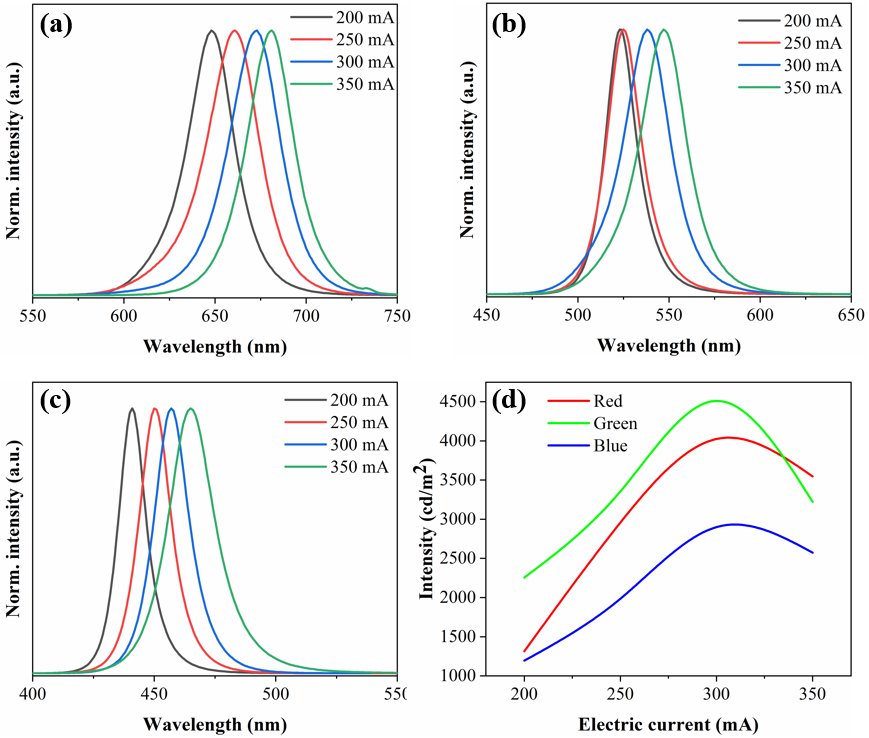


**Figure S14:** PL spectra changing with the operating current of (a) the red LED, (b) the green LED, and (c) the blue LED; (d) The light intensity changing with the operating current of the LEDs.


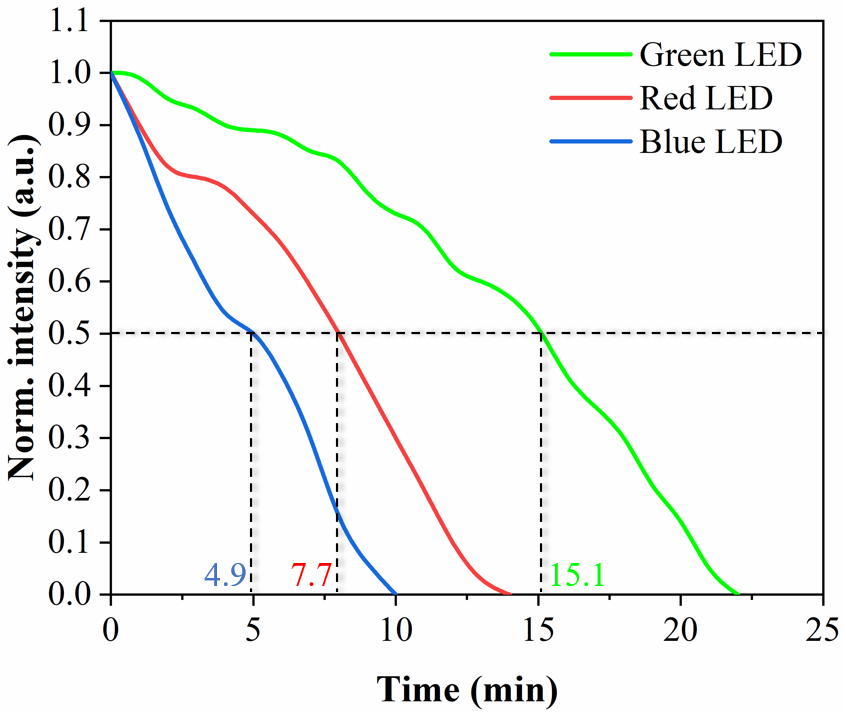


**Figure S15:** The effective operational lifetime of the three color converted LEDs.

**Table S1:** The weight percentage and atomic number percentage of the elements in each sample.

| CsPbBr_x_Cl_3-x_ | | | CsPbBr_3_ | | | CsPbBr_x_I_3-x_ | | |
| --- | --- | --- | --- | --- | --- | --- | --- | --- |
| Element | wt% | at% | Element | wt% | at% | Element | wt% | at% |
| C | 11.21 | 45.60 | C | 17.18 | 49.87 | C | 24.85 | 62.48 |
| O | 0.62 | 1.88 | O | 14.07 | 30.68 | O | 11.86 | 22.39 |
| Cl | 19.17 | 26.41 | Cs | 12.87 | 3.38 | I | 25.63 | 6.10 |
| Cs | 16.95 | 6.23 | Pb | 31.00 | 5.22 | Cs | 11.18 | 2.54 |
| Pb | 31.78 | 7.49 | Br | 24.88 | 10.86 | Pb | 15.12 | 2.20 |
| Br | 20.27 | 12.39 | Total | 100.00 | 100.00 | Br | 11.36 | 4.29 |
| Total | 100.00 | 100.00 |  |  |  | Total | 100.00 | 100.00 |

**Table S2:** The fitting equations and the corresponding fitting parameters of fluorescence intensity and fluorescence lifetime, where I(t) is the fluorescence intensity changing with time, R^2^ is the decision coefficient, and τ_avg_ is the average fluorescence lifetime.

|  | R-PQDs | A-R-PQDs | G-PQDs | A-G-PQDs | B-PQDs | A-B-PQDs |
| --- | --- | --- | --- | --- | --- | --- |
| I_0_ | 6.395 | 59.519 | 6.099 | 12.144 | 2.067 | 5.126 |
| A_1_ | 6057.078 | 684.886 | 4470.733 | 1347.729 | 3296.799 | 3016.537 |
| t_1_[ns] | 20.178 | 6.990 | 2.010 | 2.840 | 1.363 | 1.075 |
| A_2_ | 3572.713 | 4936.499 | 5187.528 | 6255.789 | 4933.776 | 4939.424 |
| t_2_[ns] | 40.421 | 29.360 | 6.286 | 12.161 | 5.782 | 6.631 |
| A_3_ | 76.508 | 4015.822 | 262.280 | 2028.117 | 1499.789 | 1848.767 |
| t_3_[ns] | 249.122 | 89.167 | 33.975 | 34.181 | 16.112 | 20.120 |
| R^2^ | 0.99958 | 0.99946 | 0.99959 | 0.99961 | 0.99947 | 0.99951 |
| τ_avg_[ns] | 45.6 | 71.3 | 10.4 | 22.2 | 9.7 | 13.2 |
| Fitting equation | $I\left( t \right)=A_{1}ⅇ^{-\frac{t}{t_{1}}}+A_{2}ⅇ^{-\frac{t}{t_{2}}}+A_{3}ⅇ^{-\frac{t}{t_{3}}}+I_{0}$ | | | | | |
| Calculation equation of τ_avg_ | $\tau_{avg}=\frac{A_{1}t_{1}^{2}+A_{2}t_{2}^{2}+A_{3}t_{3}^{2}}{A_{1}t_{1}+A_{2}t_{2}+A_{3}t_{3}}$ | | | | | |

**Table S3:** Color coordinates in CIE 1931, tristimulus values (TSV), and color coordinates CIE 1976 uniform color space (UCS) of the three-primary-color LEDs.

| Color | CIE 1931 | | TSV | | | CIE 1976 UCS | |
| --- | --- | --- | --- | --- | --- | --- | --- |
|  | x | y | X | Y | Z | u’ | v’ |
| Red | 0.7008 | 0.2863 | 18484.6 | 8286.7 | 43.2 | 0.5568 | 0.5119 |
| Green | 0.1336 | 0.7777 | 4752.9 | 40158.8 | 6176.1 | 0.0443 | 0.5801 |
| Blue | 0.1621 | 0.0210 | 32072.6 | 5293.3 | 174958.3 | 0.2215 | 0.0644 |

**Table S4:** Color coordinates of three-primary-color LED devices, NTSC standard, and Rec.2020 standard.

| Color | LEDs | | NTSC | | Rec.2020 | |
| --- | --- | --- | --- | --- | --- | --- |
|  | x | y | x | y | x | y |
| Red | 0.7008 | 0.2863 | 0.670 | 0.330 | 0.7079 | 0.2920 |
| Green | 0.1336 | 0.7777 | 0.210 | 0.710 | 0.1702 | 0.7965 |
| Blue | 0.1621 | 0.0210 | 0.140 | 0.080 | 0.1314 | 0.0459 |

**Note 1: Experimental section**

*Chemicals and Reagents:* Cesium carbonate (Cs_2_CO_3_, 99.9%), oleic acid (OA, ≥99%), oleylamine (OAm, ≥98%), octadecenes (ODE, 90%), lead chloride (PbCl_2_, 99.999%), lead bromide (PbBr_2_, 99.999%), and lead iodide (PbI_2_, 99.999%) were purchased from Sigma-Aldrich. Macroporous strong basic anion exchange resin, PDMS, qualitative filter paper, sodium chloride (NaCl, AR), sodium bromide (NaBr, AR), sodium iodide (NaI, AR), n-hexane (AR, ≥95%), cyclohexane (AR, ≥95%), toluene (AR, ≥95%), and p-xylene (AR, ≥95%) were purchased from Shanghai Aladdin Company. All reagents were used without further purification.

*Preparation and Purification of CsPbBr_3_ PQDs:* Firstly, the cesium oleate precursor was prepared. 0.4 g Cs_2_CO_3_ was mixed with 1.5 mL OA and 20 mL octadecene in a three-necked flask, and the flask was heated to 120 ℃ for continuous stirring for 1 h under N_2_ atmosphere. Then the temperature was raised to 160 ℃ for 20 min, and the transparent cesium oleate precursor solution was obtained. The precursor solution has to be sealed, stored, and preheated to 120 °C before use. 0.15 g PbBr_2_ was mixed with 25 mL octadecene in a three-necked flask, and continuously stirred for 30 min under N_2_ atmosphere. After that, the temperature was raised to 120 ℃ followed by stirring for 30 min. 1 mL OA and 2 mL OAm were mixed into a syringe, then quickly injected into the three-necked flask, stirred for a while to get a clear solution. Then the temperature was rapidly raised to 180 ℃, and 2 mL of cesium oleate precursor solution was quickly injected into the three-necked flask. After 3 seconds of reaction, the solution was immediately transferred to ice water and cooled until the temperature of the solution dropped below 30 ℃. The product was centrifuged in a centrifuge at 10000 rpm/min for 8 min. After centrifugation, the precipitate was dispersed in cyclohexane. The precipitate was completely dispersed by ultrasound, and then centrifuged at 6000 rpm/min for 8 min. After centrifugation, the supernatant was taken and stored. Finally, the concentration of the CsPbBr_3_ QD solution produced by the reaction is about 9 mmol/L.

*Type Conversion of Anion Exchange Resins:* 10 g of deionized water was placed in a reagent flask and 3.59 g NaCl (9.05 g NaBr or 18.4 g NaI) was added in, and NaCl (NaBr or NaI) was completely dissolved at 500 rpm/min for 5 min to make a saturated solution. 1 g of anion exchange resin was added into NaCl (NaBr or NaI) saturated solution. After continuous stirring for 6 h, the anion exchange resin was removed through filtration. Repeat the above steps again to convert the resin into Type-Cl (Type-Br or Type-I). The filtered anion exchange resin was put into a drying oven at 60℃ for 1 h to dry completely, and finally was stored in a reagent bottle for further use.

*Preparation of CsPbBr_x_I_3-x_ and CsPbBr_x_Cl_3-x_ PQDs:* The dried anion exchange resin containing different halogen ions was mixed with the cyclohexane solution of CsPbBr_3_ PQDs at the ratio of 0.05 g/mL. The solution was continuously stirred at 500 rpm/min according to the predetermined reaction time, and then the solution was removed through filtration. The PQDs solution was placed in a centrifuge tube and centrifuged at 6000 rpm/min for 5 min. After that, the supernatant was taken and stored.

*Preparation of Color Converted LEDs:* The PDMS and curing agent were mixed with the mass ratio of 10 : 1, and then the appropriate amount of red, green, and blue PQDs solution was added in, respectively. Place the mixed solution in a mold and keep on a heating table at 80 ℃ for 2 h curing. The cured three-primary-color PQDs layer were attached to the 365 nm UV lamp chip, and the appropriate voltage and current were adjusted after correct wiring.

*Performance Test of* *the Color Converted LEDs:* The operating voltage of the LED was adjusted and fixed to 3.4 V, and the operating current was set to 200 mA. The relative position of the LED and the luminance meter was fixed with the field of view of 0.2°. The brightness change of the LED was continuously monitored when the current was slowly increased to 350 mA. The spectrum was tested when the current was 250 mA, 300 mA, and 350 mA, respectively. After the above test was completed, the current was set back to 200 mA, and the brightness change of the LED was continuously tested. The above test was repeated three times for three-primary-color LEDs, respectively.

**Reference**

[S1] Q. Jing, M. Zhang, X. Huang, et al., “Surface passivation of mixed-halide perovskite CsPb(Br_x_I_1−x_)_3_ nanocrystals by selective etching for improved stability,” *Nanoscale*, vol. 9, no. 22, pp. 7391-7396, 2017.
